# A Nonluminescent and Highly Virulent *Vibrio harveyi* Strain Is Associated with “Bacterial White Tail Disease” of *Litopenaeus vannamei* Shrimp

**DOI:** 10.1371/journal.pone.0029961

**Published:** 2012-02-27

**Authors:** Junfang Zhou, Wenhong Fang, Xianle Yang, Shuai Zhou, Linlin Hu, Xincang Li, Xinyong Qi, Hang Su, Layue Xie

**Affiliations:** 1 Key Laboratory of Marine and Estuarine Fisheries Resources and Ecology, East China Sea Fisheries Research Institute, Chinese Academy of Fisheries Science, Shanghai, China; 2 Aquatic Pathogen Collection Center of Ministry, Shanghai, China; 3 Shanghai Animal Disease Control Center, Shanghai, China; Cairo University, Egypt

## Abstract

Recurrent outbreaks of a disease in pond-cultured juvenile and subadult *Litopenaeus vannamei* shrimp in several districts in China remain an important problem in recent years. The disease was characterized by “white tail” and generally accompanied by mass mortalities. Based on data from the microscopical analyses, PCR detection and 16S rRNA sequencing, a new *Vibrio harveyi* strain (designated as strain HLB0905) was identified as the etiologic pathogen. The bacterial isolation and challenge tests demonstrated that the HLB0905 strain was nonluminescent but highly virulent. It could cause mass mortality in affected shrimp during a short time period with a low dose of infection. Meanwhile, the histopathological and electron microscopical analysis both showed that the HLB0905 strain could cause severe fiber cell damages and striated muscle necrosis by accumulating in the tail muscle of *L. vannamei* shrimp, which led the affected shrimp to exhibit white or opaque lesions in the tail. The typical sign was closely similar to that caused by infectious myonecrosis (IMN), white tail disease (WTD) or penaeid white tail disease (PWTD). To differentiate from such diseases as with a sign of “white tail” but of non-bacterial origin, the present disease was named as “bacterial white tail disease (BWTD)”. Present study revealed that, just like IMN and WTD, BWTD could also cause mass mortalities in pond-cultured shrimp. These results suggested that some bacterial strains are changing themselves from secondary to primary pathogens by enhancing their virulence in current shrimp aquaculture system.

## Introduction

*Litopenaeus vannamei* (*L. vannamei*) shrimp is the most extensively cultivated species worldwide for its high-yield and low-demand for concentration of salt. But with the continual expanding and intensifying of aquaculture, more and more serious viral diseases are emerging such as white spot syndrome virus (WSSV), infectious myonecrosis virus (IMNV) and *Penaeus vannamei* nodavirus (PvNV). Among these, IMNV and PvNV were documented to be causative pathogens of “white tail disease” (WTD)-like disease in marine shrimp following the *Macrobrachium rosenbergii* nodavirus (MrNV) identified in freshwater prawn [1]–[3]. The two viruses both primarily targeted the skeletal muscle and resulted in very similar gross signs (a white or opaque tail) and histopathological changes (focal to extensive areas of muscle necrosis and the formation of prominent lymphoid organ spheroids) in Penaeid shrimp. During or soon after stressful events, outbreaks of IMN are usually accompanied by high mortalities (PvNV is less virulent than IMNV) [3], [4]. Since such viral diseases are significant in shrimp aquaculture, more research attention has been paid to the viral diseases and less to the bacterial. However, with increasing global-warming and intensive aquaculture, bacterial diseases especially vibrioses are becoming another important threatening to the sustainable development of the Penaeid shrimp aquaculture industry [5].

In shrimp aquaculture system, vibrios are among the normal bacterial flora of cultural populations and the habitats [6], [7], from which *Vibrio harveyi*, *V. alginolyticus* and *V. parahaemolyticus* are most frequently isolated [8]–[12]. As opportunistic pathogens, they may lead to mortality of affected aquatic animals due to stressful events, such as sudden changes in temperature [5], [13] and salinity. Among these vibrios, *V. harveyi* is one of the most important pathogens, capable of causing devastation to diverse ranges of marine invertebrates including Penaeid shrimp [14]–[18]. In the past two decades, mass mortalities of Penaeid shrimp resulted from *V. harveyi* infections were frequently reported in hatcheries and grow-out ponds [10], [11], [19]–[22]. Notably, these pathogenic *V. harveyi* strains were generally luminescent.

In present study, a highly virulent *V. harveyi* strain HLB0905 was identified as the etiologic pathogen of bacterial white tail disease (BWTD) through microscopical examination, sequence analysis, bacterial isolation and challenge test. Interestingly, the strain not only mainly caused “white tail” in *L. vannamei* shrimp, but also was nonluminescent.

## Results

### Gross signs of affected shrimp

**Figure 1:**
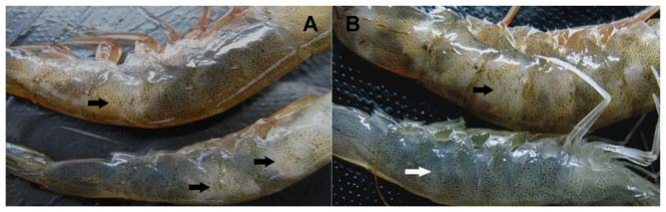
Gross signs of diseased *L. vannamei* shrimp. (A) Gross signs of *L. vannamei* shrimp naturally occurred in the farm. Focal to extensive whitish muscles in the tail (black arrows) with (top) or without (bottom) red discoloration in the body and appendages (B) Gross signs of *L. vannamei* shrimp laboratory-infected with the *V. harveyi* strain HLB0905. Compared to normal shrimp (bottom, white arrow), the infected shrimp exhibited an extensive whitish or opaque appearance in the tail (top, black arrow).

Soon after being reared in the indoor tank, many of the Penaeid shrimp, apparently healthy previously, started to show WTD-mimicking gross signs, which was characterized by focal to extensive areas of whitish muscle, particularly in the distal abdominal segments, with or without a red discoloration in the body and appendages (Fig. 1A). One day later, perhaps due to a combination of the disease and sudden stresses such as collection by cast-netting and transportation, death occurred with a sudden high mortality. Six d later, the cumulative mortality of shrimp reached up to 76%.

### Histopathological and ultrastructural analysis

**Figure 2:**
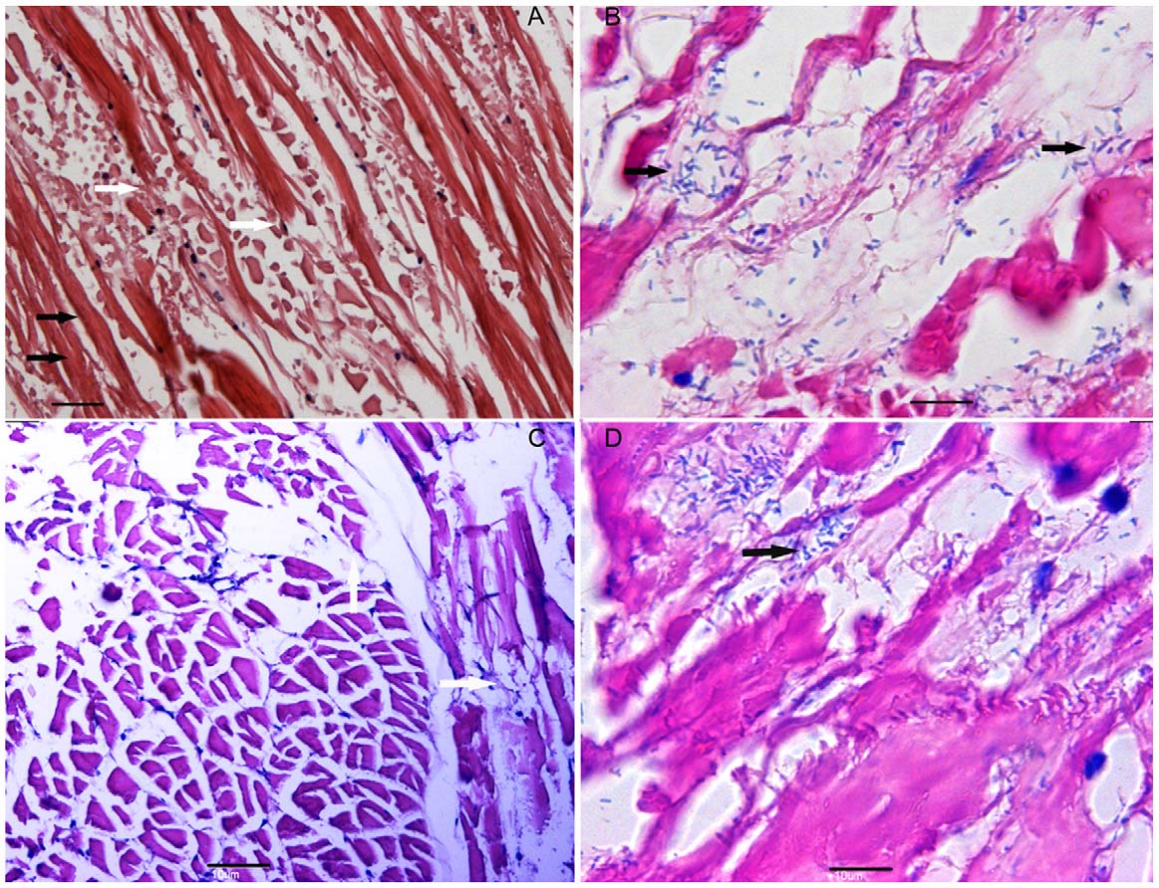
Histopathological changes in whitish muscles. (A and C) Normal (black arrows) and broken muscle fibers (white arrows). (B and D) Coagulative to liquefactive muscle necrosis and infiltration of a great number of rod-shaped bacteria (black arrows). Tissue A and B were sampled from *L. vannamei* shrimp with a WTD-like disease in the farm, while tissue C and D were sampled from *L. vannamei* shrimp laboratory-infected with the *V. harveyi* strain HLB0905(A: bar = 35 µm; B, C and D: bar = 10 µm).

**Figure 3:**
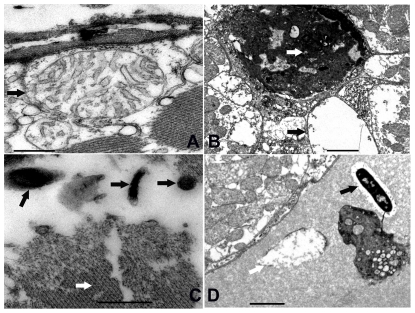
Ultrastructural changes in whitish muscles. (A) The mitochondrial membrane was broken (black arrow, bar = 0.5 µm). (B) The nuclear pyknosis (white arrow) and fiber cell vacuolation (black arrow, bar = 2 µm). (C) Damaged myofibrils (white arrow shows normal morphology of myofibrils) and invasion of bacteria (black arrow, bar = 0.5 µm). (D) Damaged fiber cells (white arrow shows damaged mitochondria) and infiltration of *Vibrio*-like bacteria (black arrow) (bar = 2 µm). All the tissues were sampled from *L. vannamei* shrimp with a WTD-like disease in the farm.

Histopathological analysis showed that muscle fibers composing the whitish tail muscle were damaged in different degrees with focal to extensive fiber necrosis in both naturally- and artificially-infected *L. vannamei* shrimp (Fig. 2). The data indicated that the opaque or whitish appearance of diseased shrimp was due to muscle necrosis. Furthermore, an electron microscopical analysis showed that fiber cells composing the whitish muscle were damaged, including nuclear pyknosis, cell vacuolation, mitochondrial damage and myofibrils damaged in different degrees (Fig. 3). Notably, light and electron microscopical analysis both demonstrated that there were lots of rod-shaped bacteria (Fig. 2B, 3C and 3D) with a flagellum at one end (Fig. 3D) infiltrating in these necrotic muscles, and except for these, there were not any kinds of microorganisms such as viruses and parasites being observed over a large number of ultrathin sections cut consecutively. These analyses suggested that a *Vibrio*-like bacterium may be associated with the WTD-like disease.

### Bacteria isolation and identification

**Figure 4:**
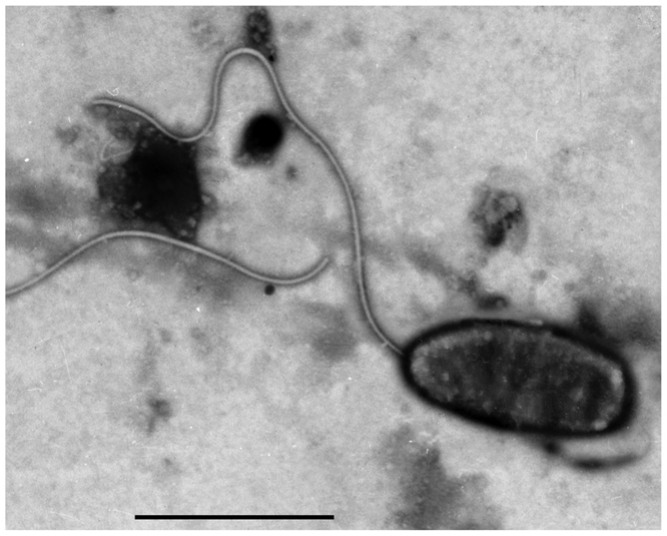
Morphology of isolated *V. harveyi* strain HLB0905 (bar = 2 µm).

Twenty-four h post incubation of muscle smears, lots of colonies with the same phenotypes (i.e. round, milky and nonluminescent) appeared on the plates. All of the colonies analyzed were able to grow on thiosulfate citrate bile salts sucrose (TCBS) agar. They were Gram-negative, sensitive to the vibriostatic agent O/129 at 150 mg, and *V. harveyi* hemolysin-positive. All of these characteristics were in accordance with those owned by *V. harveyi*. Moreover, by negative staining electron microscopy, the *V. harveyi* isolate (designated as the strain HLB0905) was a short rod-shaped bacterium with a flagellum at one end (Fig. 4).

### PCR detection and sequence analysis

**Figure 5:**
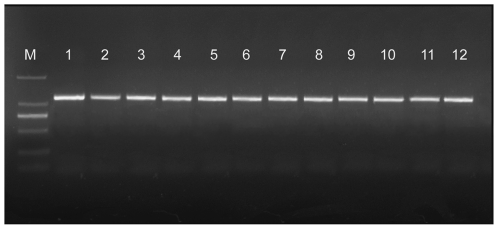
PCR products amplified from the *V. harveyi* hemolysin gene. These tissues were respectively sampled from four *L. vannamei* shrimp with a WTD-like disease in the farm. M: DL 2000 marker (Takara, Japan); 1–4, whitish muscle; 5–8, hemolymph; 9–12, hepatopancreas.

**Figure 6:**
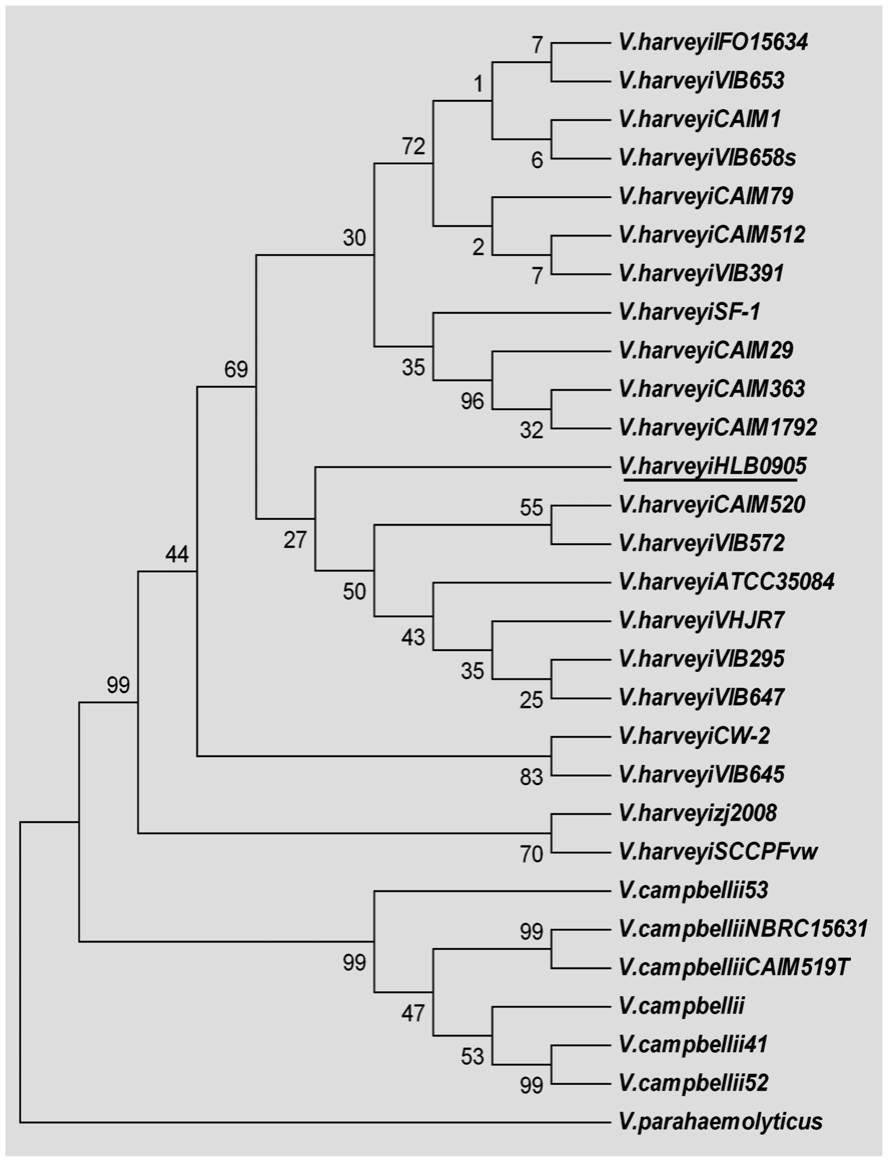
Phylogenetic tree based on the neighbor-joining method, using complete *Vibrio* hemolysin gene sequences. Bootstrap values are expressed as percentages of 1000 replications.

Based on 16S rRNA gene sequence analysis, bacteria within the whitish muscle of diseased shrimp shared the highest (99%) identity with *V. harveyi* species (Genbank accession no. **HM590018**). Since the genus *Vibrio* contains a large number of closely related bacterial species with very small difference in the sequence of their 16S rRNA gene, total genomic DNAs respectively from the whitish muscle, hepatopancreas and hemolymph of diseased shrimp were all subsequently subjected to a PCR analysis for the *V. harveyi*-specific hemolysin gene. As shown in Fig. 5, all of the samples were *V. harveyi* hemolysin-positive. Furthermore, based on the BLAST searching and phylogenetic analysis (Fig. 6), the nucleotide sequence displayed the highest homology (99%) with the reported hemolysin gene of *V. harveyi* whereas no more than 80% with that of *V. compbellii* (which generally shows the closest relationship with *V. harveyi* [23]). These data further confirmed that the *Vibrio*-like bacterium infiltrating in the whitish muscle was *V. harveyi*.

### Confirmation of the causative pathogen

In order to further identify whether the nonluminescent *V. harveyi* strain was the etiologic pathogen, bacterial challenge trials were conducted. The challenge tests showed that the HLB0905 strain was highly virulent. With a dose of 38 CFU of *V. harveyi* per shrimp, cumulative mortality of shrimp was 30% by 1 dpi, 60% by 2 dpi, and reached 100% by 4 dpi. In contrast, none of the shrimp in control groups died. Notably, most of those died in 2 dpi exhibited a focal to extensive whitish appearance in the tail (Fig. 1B). Further, the histopathological examination showed skeletal muscle necrosis and infiltration of a large number of rod-shaped bacteria (Fig. 2C and 2D). All of the observations were almost identical to those in clinical specimens. Finally, the PCR analysis of muscle DNA also indicated that the whitish muscles were strongly positive for *V. harveyi* hemolysin gene whereas muscles from shrimp in the control groups were negative (data not shown).

In conclusion, the present study demonstrated that the WTD-like disease, occurred in pond-cultured *L. vannamei* shrimp in Hainan Province, was caused by the nonluminescent but highly virulent *V. harveyi* strain HLB0905. Thus, to differentiate from other diseases with the similar sign of “white tail” but of non-bacterial origin such as IMN, WTD and PWTD, the present disease was named as “bacterial white tail disease (BWTD)”.

## Discussion

*Vibrio* species comprise the most frequently encountered bacterial pathogens of cultivated shrimp, and *V. harveyi* is amongst the most isolated [8]–[12]. Many *Vibrio* virulence factors including various enzymes (e.g., proteases and lipases), siderophores and proteinaceous toxins have been identified. They were considered to be the important determinants of iron binding and biofilm forming whereas to be regulated by quorum sensing [24]–[26]. However, since the composition and expression level of genes encoding these virulence factors generally vary with *Vibrio* spp. and strains, there is a large degree of virulence-variation and genetic diversity among different *V. harveyi* isolates. Some isolates couldn't cause shrimp mortality at high doses (10^5^–10^7^ cells per g shrimp body weight); while others were lethal at 10^3^ per g shrimp body weight or less [27]. Notably, our isolate *V. harveyi* HLB0905 was able to cause 60% of the challenged shrimp to die during 48 h at a dose of 38 CFU per shrimp, which indicated that the isolate HLB0905 was highly virulent. It has been demonstrated that *V. harveyi* can change its virulence through horizontal gene transfer or quorum sensing [27]–[30]. For example, via transferring virulent genetic elements, bacteriophages may either promote the production of an existing toxin or somehow induce the production of a new one. As a result, the phenotype of a *V. harveyi* strain was converted from non-virulent to virulent or was enhanced from less-virulent to highly virulent [27], [31], [32]. Furthermore, in some cases, the pore-forming activity of haemolysin is not restricted to erythrocytes, but extends to a wide range of other cell types, and enhances virulence by causing tissue damage [33]. In the present study, *V. harveyi* HLB0905 caused both severe muscle tissue necrosis and mass mortalities in the affected shrimp. Whether the *V. harveyi* HLB0905 has enhanced its virulence via one of the cases above or a combination of the several ones is being studied.

During the investigation, we found that occurrence of BWTD was closely climate- and aquaculture density-linked. For example, besides Qionghai district of Hainan Province, the *V. harveyi* HLB0905 was isolated and identified in Fuding district of Fujian Province (data not shown), and the average water temperature in these two provinces are generally greater than 29°C in summer. In contrast, in other provinces mainly with lower water temperature, the disease hasn't yet been detected. In fact, many researches [27]–[29], [34] have showed that the cell density of *V. harveyi* can be promoted by sudden changes in environments and conditions in aquaculture such as temperature, salinity, nutrient concentration, niche switching and host animal density. The changed cell density then made horizontal gene transfer easier and/or induced quorum-sensing regulation. Moreover, selection pressure exerted by dramatic environmental changes generally perturbs. Many of the perturbed genes and regulatory networks were demonstrated to be preferentially modulating virulence mechanisms [35]. Obviously, virulence of *V. harveyi* strain was closely related to environmental factors, suggesting that environmental factors need to be deeply concerned in shrimp aquaculture.

Based on our observations, the typical sign of whitish appearance in *L. vannamei* shrimp caused by *V. harveyi* was very similar to that caused by some viruses such as IMNV [2] and PvNV [3] or some parasites such as microsporidium [36]. In order to distinguish the causative agent of the present BWTD in all these potential pathogens, all of the tissue sections from the whitish muscle were carefully examined for these pathogens besides vibrios by the light and electron microscopy and PCR method (IMNV- and PvNV-negative by RT-PCR, data not shown). The examinations showed that the nonluminescent *Vibrio* strain was the only pathogen in the whitish muscle, indicating that present BWTD was caused by *V. harveyi*. The results suggested that, under current management, some vibrios have changed themselves from secondary to primary pathogens by enhancing their virulence.

In conclusion, via isolation, subculture, reinfection and reisolation as well, the bacterium strain HLB0905 isolated from whitish muscle was identified as the causative agent of BWTD in the pond-cultured *L. vannamei* according to Koch's Postulates. The nonluminescent *V. harveyi* HLB0905 (its luminescent isogenic counterpart should be more virulent [37], [38]) is highly pathogenic and generally causes epizootics with high mortalities in pond-cultivated Penaeid shrimp populations, especially following stressful events. Therefore, in practical terms for shrimp farmers, taking adequate prophylaxes such as the use of immunostimulants or probiotic bacteria to alleviate stresses and using pathogen-specific methods such as PCR to ensure the accurate diagnosis of WTD-like diseases would benefit to the prevention of severe disease outbreaks and economic losses in shrimp aquaculture industry [2], [9], [23], [39].

## Materials and Methods

### Shrimp and tissue sections

*L. vannamei* shrimp (10 g mean weight), with or without an appearance of white tail, were collected from a commercial shrimp farm in Hainan Province in China, which were suffering from an outbreak of WTD-like disease. Fed with commercial diet at approximately 5% of body weight with a 50% water exchange and air supply daily at 29±1°C, all the Penaeid shrimp were temporarily reared in an indoor tank. Meanwhile, moribund shrimp were picked out in time for analysis. Ultrathin sections were prepared from whitish muscles of diseased shrimp for light and electron microscopical examination. For light microscopy, the whitish muscle was fixed in 10% formaldehyde solution, embedded with paraffin and stained with hematoxylin and eosin [40]. For transmission electron microscopy, whitish muscle from the same shrimp was fixed in 2.5% glutaraldehyde in 0.1 M PBS (pH 7.4) for 2 h at 4°C, followed by in 1% osmium tetroxide for 2 h, embedded in Spurr's resin and stained with uranyl acetate and lead citrate.

### PCR detection and DNA sequencing

For PCR detection, 10 moribund shrimp (6 for 16S rRNA analysis and 4 for hemolysin gene detection) with the WTD-mimicking gross signs were randomly collected from the farm. The whitish muscle, hepatopancreas and hemolymph were respectively sampled, followed by preparation of total genomic DNA using commercially available TIANamp Marine Animals DNA Kit (Tiangen, China). Two pairs of primers, one for evolutionarily conserved 16S rRNA gene of bacteria [41] and the other for the whole *V. harveyi* hemolysin (*Vh*) gene (Table 1), were respectively synthesized. The amplification condition for both the two genes was pre-denaturation at 95°C for 30 s, 30 cycles of 95°C for 30 s, 54°C for 50 s, 72°C for 105 s, followed by elongation at 72°C for 8 min. The PCR products were subsequently sequenced.

**Table 1:** Primers used in this study.

Primer	Sequence (5′to 3′)	Amplicon Size	Reference
*Vh* F	ATGAATAAAACTATTACGTT	1254 bp	[26]
*Vh* R	GAAAGGATGGTTTGACAATT		
IMNV F (1-step)	CGACGCTGCTAACCATACAA	328 bp	[4]
IMNV R (1-step)	ACTCGGCTGTTCGATCAAGT		
IMNV F (2-step)	GGCACATGCTCAGAGACA	139 bp	[4]
IMNV R (2-step)	AGCGCTGAGTCCAGTCTTG		
PvNV F (1-step)	CTGTCTCACAGGCTGGTTCA	339 bp	[3]
PvNV R (1-step)	CCGTTTGAATTTCAGCAACA		
PvNV F (2-step)	CAAAACTGTGCCTTTGATCG	246 bp	[3]
PvNV R (2-step)	GCCTTATCCACACGAACGTC		
16S rRNA F	AGA GTT TGA TCC TGG CTC AG		[41]
16S rRNA R	AAG GAG GTG ATC CAG CC		

Homology study was carried out using BLAST Searching (NCBI). The sequences were aligned using ClustalX, and phylogenetic trees were constructed by the neighbor-joining method [42]. The robustness of each topology was checked by 1000 bootstrap replications. Trees were drawn by using MEGA version 4.0.

### Bacteria isolation and cultivation

Whitish muscles were aseptically sampled from freshly collected moribund *L. vannamei* shrimp suffering from a WTD-like disease in the farm. Muscle blocks from different shrimp were respectively touched and streaked on tryptic soy agar plates (TSA, supplemented with 2% NaCl [w/v]) followed by incubation at 29°C for 24 h. Five colonies were selected randomly from each plate respectively for examination of some biochemical characteristics, PCR detection of *V. harveyi* hemolysin gene (Table 1) and microscopical analysis. Identified colonies were subcultured and stored in deep tube TSA with 2% NaCl as stocks.

### Bacterial challenge test

Apparently healthy subadult *L. vannamei* shrimp (10 g mean weight) were collected from another farm, where WTD-like disease had never occurred. Fed with commercial diet at approximately 5% of body weight with a 50% water exchange and air supply daily at 29±1°C, these shrimp were cultured temporarily in indoor cement tanks for 7 days before they were used for bacterial challenge tests. For challenge trials, 45 shrimp, free from IMNV and PvNV by RT-PCR (Table 1), were randomly selected and put into three fiber tanks, each containing 15 shrimp and 90 L of seawater at 29±1°C. The *V. harveyi* cells, pure cultured in tryptic soy broth (TSB) with 2% NaCl, were collected and diluted with 0.01 M PBS (pH 7.4). For each treatment, each shrimp received 50 µl of an aliquot of *V. harveyi* (average 38 CFU/shrimp, decided by a pre-treatment test) by injected intramuscularly into the abdominal segment using a syringe with a 29-gauge needle. Shrimp in the control group were injected with an equal volume of 0.01 M PBS (pH 7.4). Animals were observed for the sign of “white tail” and the mortality after injection.
